# The Association of Serum TNF-α Levels and Blood Multi-Elements Modified by TNF-α Gene Polymorphisms in Metal Industrial Workers

**DOI:** 10.3390/ijerph16214079

**Published:** 2019-10-23

**Authors:** Tzu-Hua Chen, Joh-Jong Huang, Wei-Shyang Kung, Su-Shin Lee, Hung-Yu Sun, Hung-Yi Chuang

**Affiliations:** 1 Department of Public Health, College of Health Sciences, Kaohsiung Medical University, Kaohsiung 80708, Taiwan; licavona@gmail.com; 2 Department of Family Medicine, Kaohsiung Municipal Ta-Tung Hospital, Kaohsiung 80145, Taiwan; 3 Department of Family Medicine, Kaohsiung Medical University Hospital, Kaohsiung 80708, Taiwan; jjhua511227@gmail.com; 4 Department of Pediatrics, National Cheng Kung University Hospital, Tainan 70101, Taiwan; Kungweishyang@outlook.com; 5 Center for Stem Cell Research, Kaohsiung Medical University, Kaohsiung 80708, Taiwan; k831702000@gmail.com; 6 Department of Family Medicine, Changhua Christian Hospital, Changhua 50006, Taiwan; b87205216@yahoo.com.tw; 7 Department of Environmental and Occupational Medicine, Kaohsiung Medical University Hospital, Kaohsiung 80708, Taiwan

**Keywords:** TNF-α, single nucleotide polymorphism (SNP), mixed-effect model, multi-elements, metal workers

## Abstract

Health of the metal industrial workers should be a noteworthy issue due to the hazard of chronic exposure to metals or toxic elements. The interactions among multiple elements are sophisticated and may differ from person to person. Tumor necrosis factor-α (*TNF-α*) *gene* polymorphisms were supposed to be involved with the interactions because TNF-α plays an important role in inflammation, a mechanism by which toxic elements cause threats to human health. This research aimed to analyze the influence of *TNF-α gene* polymorphisms and multi-elements on serum TNF-α level. Blood multi-elements concentrations (lead, cadmium, arsenic, selenium, cobalt, copper, and zinc), serum TNF-α level, and *TNF-α* single nucleotide polymorphisms (SNPs), including −238G > A (rs361525), −308G > A (rs1800629), −857C > T (rs1799724), −863C > A (rs1800630), and −1031T > C (rs1799964), were measured in 462 metal industrial workers. We applied mixed-effect models to analyze the interactions among multi-elements and TNF-α SNPs. Blood concentration of all elements were positively associated with serum TNF-α level, and the effects may be modified by TNF-α gene polymorphisms. Our study revealed that TNF-α −308A/A and −1031C/C may be susceptible genotypes, and thus we suggest that those workers should take preventive measures against metal toxicity.

## 1. Introduction

Workers in metal industries are likely to be exposed to multiple metals. Lead (Pb), cadmium (Cd), and arsenic (As) were reported to be the main hazardous metals/metalloids in heavy industries. They cause severe damage to many target organs in human bodies through the mechanism of inflammation, production of oxidative stress, and interference with essential elements. The interactions among these toxic metals/metalloids are extremely complicated and some effects which have not been observed in single constituent exposure may occur [1,2].

On the other hand, selenium (Se), cobalt (Co), copper (Cu), and zinc (Zn) are essential trace elements which play crucial roles in maintaining normal physiological function in human bodies, but they may cause a threat to human health beyond tolerable serum concentrations [3,4,5,6]. Under the exposure to multiple metals/metalloids which may compete with or regulate the function of the essential elements in human bodies, they may cause additive, synergistic, or antagonistic interactions, or even new effects may occur. Some animal studies addressing these sophisticated interactions have been published [7], but the true kinetic interactions in human beings still remain unclear.

One of the mechanisms of how toxic elements exert a negative effect on health is through inflammation. Tumor necrosis factor-α (TNF-α), possessing a wide range of biological activities, is known to be an important mediator of local and systemic inflammation, and immune response [8]. The main source of TNF-α is activated macrophages and monocytes, moreover, some other cells, such as lymphocytes, polymorphonuclear leukocytes, keratinocytes, and tumor cells, may also secrete TNF-α [9]. Owing to the dual properties of pro- and anti- inflammatory cytokines, TNF-α plays a role in defensive responses, but may cause damage to organs once the balance is broken [10]. Overexpression of TNF-α is considered to contribute to various diseases, such as inflammatory bowel disease, rheumatoid arthritis, and sepsis [10].

The TNF gene cluster is located on human chromosome 6p21. Several single nucleotide polymorphisms (SNPs) have been identified in the human TNF gene promoter [11]. The SNPs vary among different ethnic groups and they have been reported to be associated with susceptibility to some diseases, such as infection, sepsis, systemic lupus erythematosus (SLE), rheumatoid arthritis (RA), Crohn’s disease, coronary heart disease (CHD), and cancer [11,12,13]. Five SNPs within the TNF promotor region (−238, −308, −857, −863, −1031) were considered to influence TNF-α production, and thus they have been suggested to influence the susceptibility to some diseases [13,14,15].

Most metal industrial workers are exposed to various metals/metalloids and elements simultaneously in their working environment. The complicated interactions and impacts on health are major issues but have not been studied thoroughly. The aim of our research is to evaluate the interactive effects between multi-elements. Moreover, we also investigate how TNF-α gene polymorphisms modify the influence of multi-elements on serum TNF-α level.

## 2. Materials and Methods 

### 2.1. Participants and Health Examinations

We collected the data of 462 metal industrial workers who received annual health examination in Kaohsiung Medical University Hospital, a medical center in southern Taiwan. All procedures were approved by the Kaohsiung Medical University Hospital Institutional Review Board (approval number: KMUHIRB-E(I)-20150259), and written informed consent was obtained from all participants. The content of health examinations consisted of body mass index (BMI) and blood pressure measurement, personal history inquiry (including medical history, working history, and the habit of smoking or drinking alcohol), physical examination, and blood tests. Blood sampling for biochemical analyses included white blood cell (WBC) counts, red blood cell (RBC) counts, hemoglobin, platelet counts, aspartate aminotransferase (AST), alanine aminotransferase (ALT), total cholesterol, triglyceride, uric acid, fasting blood sugar, and creatinine. In addition, the blood samples were sent for TNF-α genotype analysis and measurement of blood multi-elements concentration (Pb, Cd, As, Se, Co, Cu, and Zn) and serum TNF-α level. All blood samples were analyzed in the central laboratory in Kaohsiung Medical University Hospital.

### 2.2. Analyses of Blood Multi-Elements and Serum TNF-α Level

Venous blood samples obtained in annual health examinations were stored at 4℃, and then were placed to room temperature prior to analysis. We set the mixed solution with 0.2 % ammonia solution, 0.1% Triton X-100, and 0.3% HNO_3_ for dilution solution. Then, the blood samples were analyzed by Inductively Couple Plasma Mass Spectrometry (ICPMS) of Thermo Scientific XSERIES 2 (Standard glass nebulizer kit 4600294-3; Spray chamber 1600061; Ni Sample cone 3600812; Ni Skimmer cone (Xt) 3600811) (Thermo Fisher Scientific Inc. Bremen, Germany). We checked whole blood concentrations of Pb, Cd, As, Se, Co, Cu, and Zn. The process of analyses was modified from the method of Ebba Barany in 2007 [16]. We conducted quality accuracy (QA) and quality control (QC) to make sure precision and accuracy. QA was to analyze standard reference materials (SRMs). To ensure the consistence of laboratory test, we took random SRMs to conduct repeated analysis, and each result had to fit the curve between 90% and 110%. QC was to make sure the stability of system by triple testing the SRM sample, which coefficient of variance (CV) should be less than 3%.

Serum samples were collected from coagulated blood. Serum TNF-α levels were measured by Enzyme-linked immunosorbent assay (ELISA) using Human TNF-alpha Quantikine ELISA Kit from R&D Systems (R&D Systems, Minneapolis, MN, USA). Three samples of known concentration were tested twenty times on one plate to assess intraassay precision, and tested in twenty separate assays to assess interassay precision. The CVs of intraassay were all less than 5%, and CVs of interassay were all less than 8%.

### 2.3. TNF-α Promotor Polymorphism Genotyping

SNPs located within the TNF-α gene were selected from the NCBI LocusLink (http://Ncbi.nlm.nih.gov/LocusLink) and HapMap database (http://hapmap.org). Genomic DNA from participants was obtained from whole peripheral blood, using standard method. TaqMan allelic discrimination assays (Applied Biosystems, Foster City, CA, USA) was used to separately analyze all the SNPs: TNF-α −238G > A (rs361525), −308G > A (rs1800629), −857C > T (rs1799724), −863C > A (rs1800630), and −1031T > C (rs1799964). The results were analyzed with a 7300 Real-time polymerase chain reaction (PCR) system (Applied Biosystems(ABI)®, Thermo Fisher Scientific, Foster City, CA, USA). Each real-time PCR was performed with a 10μL reaction volume mix fluid, containing 5μL Genotyping Master Mix, 3.87μL distilled water, 1μL DNA fluid (10 ng/μL) and 0.25μL primer-probe. Amplification reactions were performed using the following program for total 45 cycles: 50℃ for 2 minutes; 92℃ for 10 minutes; 95℃ for 15 seconds; 60℃ for 1 minute. The fluorescence level was detected by an Applied Biosystem StepOne Real-Time PCR system (ABI®, Thermo Fisher Scientific, Foster City, CA, USA). The allele frequencies were determined using ABI SDS software (Thermo Fisher Scientific, Foster City, CA, USA).

### 2.4. Statistical Analyses

The participants were categorized into quartiles according to serum TNF-α level. Group differences in the distributions of categorical variables and mean values of continuous variables were compared by the Chi-square test and one-way analysis of variance (ANOVA), respectively. We calculated the frequencies of all TNF-α genotypes, using either the Chi-square test or the Fisher’s exact test to examine deviations from Hardy–Weinberg equilibrium (HWE). The above data were analyzed using the SPSS 20 statistical package (IBM Corporation, New York, USA).

In the Pearson correlation matrix of TNF-α and seven elements, all Pearson correlation coefficients were less than 0.5, except for Zn and Cu (γ = 0.51), so we supposed that these elements were not correlated with one another. We applied mixed-effects models for multivariate analyses, in which the blood concentrations of seven elements were set as independent variables and serum TNF-α level was set as the dependent variable. Age, gender, BMI, consumption of cigarettes and alcohol, and white blood cell (WBC) counts were incorporated as covariates. Subsequently, TNF-α SNPs were also added into independent variables in further analysis to show the modification of TNF-α gene polymorphisms. In theory, there should be 3^5^ = 243 kinds of combination genotypes based on five SNPs, but there were only 36 genotypes in our participants. After excluding 12 genotypes of *n* = 1, the result was not influenced, so we remained all 36 genotypes in our analysis for fear of deleting some genotypes. Among all the genotypes, G-G/G-G/C-C/C-C/T-T was the wild type with the most subjects, and it was set as reference. Mixed-effects models were carried out using SAS 9.4 software (SAS Institute, Inc., Cary, NC, USA). Because we analyzed five SNPs at a time, we used Bonferroni’s correction for multiple testing. α level was set at 0.05, and the corrected α level would be changed to 0.01.

## 3. Results

Table 1 lists the demographic characteristics, biochemical data, and blood multi-elements concentration of all participants according to serum TNF-α quartiles. Participants in the fourth quartile serum TNF-α group had the highest age and creatinine, and most were males. On the other hand, those in the first quartile had the highest BMI and uric acid. Participants in the third and fourth quartile serum TNF-α group had higher blood concentration of Pb, Cd, As, Co, Cu, and Zn. Table 2 shows TNF-α genotypes of all participants grouped into quarters. Wild genotypes of TNF-α (−308G > A, GG) and (−857C > T, CC) are associated with lower serum TNF-α level, while wild genotypes of TNF-α (−238G > A, GG) and (−863C > A, CC) are associated with higher serum TNF-α level. The TNF-α genotype distribution is in Hardy–Weinberg Equilibrium (HWE) except for −238G > A (rs361525), which will be discussed later.

Multivariate analyses showed that age, WBC count, and blood concentration of seven elements were positively associated with serum TNF-α level, while BMI was negatively associated with serum TNF-α level (Table 3 and Table 4). With regard to gender, males had higher serum TNF-α level than females (Table 3), but the difference no longer existed after adjustment for TNF-α SNPs (Table 4). TNF-α gene polymorphisms do modify the impacts of seven elements (Pb, Cd, As, Se, Co, Cu, and Zn) on serum TNF-α level; the percentage change of serum TNF-α level by TNF-α SNPs is 4.32%, 7.71%, 7.50%, 6.58%, 9.68%, 6.75%, and 6.73%, respectively.

In Table 4, there are decreased serum TNF-α levels in the genotypes G-A/G-G/C-C/C-C/T-T (β = −13.68), G-G/G-G/C-C/C-A/T-T (β = −11.55), G-A/G-G/C-C/C-A/T-T (β = −12.48), A-A/G-G/C-C/C-A/T-T (β = −11.64), G-G/G-A/C-C/C-A/T-T (β = −8.11), and G-G/G-A/C-C/C-C/T-C (β = −5.53). On the other hand, there are increased serum TNF-α levels in the genotypes G-G/A-A/C-C/C-C/T-T (β = 14.90), G-A/G-G/C-C/C-C/T-C (β = 9.90), G-A/G-G/C-C/C-A/T-C (β = 22.23), G-G/G-A/C-T/C-C/T-C (β = 6.71), G-G/G-G/C-C/C-C/C-C (β = 6.57), G-G/G-A/C-T/C-C/C-C (β = 17.87), G-G/A-A/C-T/C-C/C-C (β = 28.78), and G-G/A-A/T-T/C-C/C-C (β = 44.98). The genotype G-A/G-G/C-C/C-C/T-T was associated with a decreased serum TNF-α level (β = −13.68, 95% confidence interval (CI): −24.85, −2.51), whereas the genotype A-A/G-G/C-C/C-C/T-T had no association (95% CI: −28.90, 2.74). Therefore, we suggest that TNF-α −238G/A heterozygous genotype may play a protective role in the workers. The genotype G-G/A-A/C-C/C-C/T-T was associated with an increased serum TNF-α level (β = 14.90), whereas the genotype G-G/G-A/C-C/C-C/T-T had no association. Therefore, we propose that TNF-α −308A/A may be a susceptible genotype. The genotypes G-G/G-G/C-T/C-C/T-T and G-G/G-G/T-T/C-C/T-T were not associated with any change of serum TNF-α level, and thus no association was observed in TNF-α −857 SNP. Nevertheless, serum TNF-α level was higher in G-G/A-A/T-T/C-C/C-C (β = 44.98) than that in G-G/A-A/C-T/C-C/C-C (β = 28.78), so we postulated that TNF-α −857 T might be a susceptible allele. The genotype G-G/G-G/C-C/C-A/T-T was associated with a decreased serum TNF-α level (β = −11.55), and thus we proposed that TNF-α −863C/A heterozygous genotype may play a protective role. The genotype G-G/G-G/C-C/C-C/C-C was associated with an elevated serum TNF-α level (β = 6.57), so we suggested that TNF-α −1031C/C may be a susceptible genotype.

## 4. Discussion

Most researches of TNF-α gene polymorphism focus on genetic susceptibility to autoimmune diseases, inflammatory diseases, and cancers. Few studies have been reported to evaluate the correlation between TNF-α gene polymorphism and susceptibility to metal toxicity in industrial workers. It has been proposed that TNF-α gene polymorphisms may be associated with lead-induced inflammatory response [17], beryllium-induced chronic beryllium disease [18], and risk of pulmonary tuberculosis in iron miners with exposure to silica dust [19]. Nevertheless, they only explore the effect of single element, not considering the interactive effects of multi-elements in the working environment.

TNF-α −238 and −308 SNPs are two of the most extensively studied polymorphisms in many diseases [20,21,22]. The genotype GG is the most frequent in both SNPs in various ethnic groups [23,24], which is consistent with our findings. In our study, we proposed that TNF-α −238 G/A might be a protective genotype and −308A/A might be a susceptible genotype. The results were in line with most prior literatures. A meta-analysis research demonstrated an association between TNF-α −238 polymorphism and SLE susceptibility. The genotype G/A was a protective factor, whereas A/A was a risk factor for susceptibility of SLE [23]. A research in Korea revealed that TNF-α −238A allele may play a protective role against various types of cancer [25]. There has been conflicting evidence whether TNF-α −308A allele is a risk factor for inflammation. TNF-α −308A allele has been reported to increase the risk of many diseases, such as cerebral malaria, systemic lupus erythematosus (SLE), rheumatoid arthritis (RA), ankylosing spondylitis, Crohn’s disease, cancer, and coronary heart disease (CHD) [12,26]. By contrast, TNF-α −308G allele was regarded as a risk factor in some diseases and cancers [27,28]. J.H. Kim et al. proposed that TNF-α −308G/G genotype may increase susceptibility to lead-induced inflammation in non-occupational population [17], but the effect of other metals in the environment was not considered, and thus the result would be different from ours.

Our results showed that TNF-α -857T allele might be associated with a hazardous trend. Meta-analyses exhibited that TNF-α −857T allele was associated with increased susceptibility to gastric cancer, type 1 diabetes, and psoriatic arthritis [29,30,31,32]. Our study revealed that TNF-α −863C/A might be a protective factor and −1031C/C might be a susceptible genotype. Prior researches demonstrated that TNF-α −863A allele may reduce the risk of chronic obstructive pulmonary disease (COPD) and asthma [33,34,35]. TNF-α −1031C allele had an association with Behcet’s disease, Crohn’s disease, and prostate cancer [36,37,38].

In our study, age and WBC counts have a positive correlation with TNF-α, whereas BMI has a negative correlation with serum TNF-α level. Previous literature has postulated that obesity is related to low-grade inflammation, which probably results in an increased serum TNF-α level [39]. However, a prospective study in Amerindian women revealed a negative correlation between TNF-α and body weight change [40]. They supposed that high TNF-α level may induce anorexia and energy expenditure, which may be explainable for body weight reduction. Hence, the correlation between BMI and TNF-α level is still equivocal.

This is the first study using mixed-effects models to analyze the interaction of TNF-α SNPs and multi-elements in working environment. A mixed-effect model has been recommended as a powerful tool in longitudinal genome-wide association study [41]. In our research, we measured each individual’s blood concentration of seven elements from the same tube of blood sample repeatedly at different time. Due to the correlation between elements, they could be regarded as correlated data by repeat measure, therefore, we applied mixed-effects models for adjusting the complex interactive effects of multi-elements. Furthermore, we analyzed the association between multiple TNF-α SNPs and serum TNF-α level to indicate how genes interact with each other. We consider it a creative method for analyzing complex gene-gene and gene-environment interactions.

Working duration not included into the variables and relatively small sample size are two of the limitations of our research. On the other hand, the TNF-α genotype distribution in our study is in HWE except for −238G > A (rs361525). Because the expected count was less than five in −238A/A genotype, we used Fisher’s exact test instead of the Chi-square test to examine deviations from HWE [42]. On the basis of our result, TNF-α -238G/A might be a less susceptible genotype, and the number of the workers with −238G/A genotype was the highest in the lowest TNF-α group (<12.84 pg/mL). We propose that the genetic distribution of workers according to TNF-α quartiles may be influenced by genetic susceptibility. Moreover, the frequency of −238A allele in our research is 0.045, which is lower than 0.071, 0.063, and 0.09 in Koreans, Italians, and blacks of South Africa, respectively [25]. We suppose that deviations from HWE may be attributable to a small sample size and few minor-allele counts [42]. Irrespective of mild decrease of the statistical power, we still obtain some important findings in this pioneering research about complex interactions.

## 5. Conclusions

The elements in our research (Pb, Cd, As, Se, Co, Cu, and Zn) have positive correlation with serum TNF-α level, and the effects may be modified by TNF-α gene polymorphisms. TNF-α −238G/A and −863C/A heterozygous genotype may play protective roles, whereas −308A/A and −1031C/C genotypes and −857T allele may be susceptible genotypes. It is noteworthy that the workers with susceptible genotype combinations may predispose to metal toxicity in their working environment. Further large-scale studies are warranted to illustrate the complicated interaction of multi-elements and genetic polymorphisms.

## Figures and Tables

**Table 1:** Demographic characteristics, biochemical data, and blood element concentration of all participants divided into quartiles according to serum Tumor necrosis factor-α (TNF-α) levels.

Variable	Total	TNF-α (pg/mL)			
	*n* = 462	<12.84	12.84 ≦ TNF-α <18.99	18.99 ≦ TNF-α <24.34	≧24.34
Gender					
Male	368 (79.7)	97 (83.6)	86 (74.8)	85 (73.3)	100 (87.0)
Female	94 (20.3)	19 (16.4)	29 (25.2)	31 (26.7)	15 (13.0)
Smoking					
Yes	146 (31.6)	40 (34.5)	32 (27.8)	33 (28.4)	41 (35.7)
No	316 (68.4)	76 (65.5)	83 (72.2)	83 (71.6)	74 (64.3)
Drinking alcohol					
Yes	9 (1.9)	1 (0.9)	0 (0)	3 (2.6)	5 (4.3)
No	453 (98.1)	115 (99.1)	115 (100)	113 (97.4)	110 (95.7)
Age (year)	40.20 ± 8.62	37.96 ± 6.79	40.92 ± 10.28	40.70 ± 8.78	41.26 ± 7.94
BMI (kg/m^2^)	24.81 ± 3.41	25.67 ± 3.45	24.54 ± 3.56	24.29 ± 3.27	24.72 ± 3.23
SBP (mmHg)	124.31 ± 16.76	122.81 ± 14.53	122.48 ± 16.13	125.98 ± 16.38	125.97 ± 19.53
DBP (mmHg)	77.72 ± 12.49	77.10 ± 12.19	76.92 ± 11.49	78.06 ± 12.15	78.80 ± 14.06
WBC (10^3^/µL)	6.83 ± 1.65	6.71 ± 1.59	6.66 ± 1.54	6.82 ± 1.61	7.12 ± 1.84
RBC (10^6^/µL)	5.14 ± 0.54	5.16 ± 0.53	5.15 ± 0.55	5.08 ± 0.54	5.16 ± 0.53
Hemoglobin (g/dL)	14.95 ± 1.42	14.94 ± 1.34	14.92 ± 1.50	14.73 ± 1.46	15.21 ± 1.38
Platelet (10^3^/µL)	255.89 ± 55.03	248.71 ± 54.03	252.18 ± 52.97	262.30 ± 51.60	260.39 ± 60.71
AST (IU/L)	24.33 ± 10.09	22.92 ± 6.52	23.22 ± 6.87	25.18 ± 12.06	26.01 ± 12.96
ALT (IU/L)	29.53±20.28	27.69 ± 15.03	26.57 ± 13.51	30.68 ± 24.99	33.19 ± 24.40
TC (mg/dL)	194.60 ± 33.94	191.71 ± 32.72	191.70 ± 34.09	198.32 ± 33.42	196.66 ± 35.42
TG (mg/dL)	128.29 ± 101.20	132.60 ± 104.03	115.94 ± 66.57	125.26 ± 89.74	139.36 ± 132.80
Uric acid (mg/dL)	6.35 ± 1.63	6.75 ± 1.68	6.07 ± 1.54	6.22 ± 1.70	6.36 ± 1.53
Sugar (mg/dL)	94.24 ± 24.24	92.18 ± 11.04	95.52 ± 30.90	98.48 ± 31.57	90.77 ± 16.02
Creatinine (mg/dL)	0.93 ± 0.23	0.84 ± 0.14	0.83 ± 0.20	0.96 ± 0.22	1.07 ± 0.26
Pb (μg/L)	67.88 ± 100.54	26.00 ± 15.34	28.17 ± 28.69	62.60 ± 73.03	155.15 ± 152.80
Cd (μg/L)	1.05 ± 0.67	0.98 ± 0.57	0.98 ± 0.60	1.05 ± 0.67	1.24 ± 0.82
As (μg/L)	6.39 ± 5.22	4.47 ± 3.15	6.05 ± 4.84	6.86 ± 6.36	8.60 ± 5.36
Se (μg/L)	255.4 ± 49.9	254.5 ± 42.9	262.7 ± 53.9	253.4 ± 51.2	250.3 ± 51.1
Co (μg/L)	0.45 ± 0.26	0.42 ± 0.17	0.43 ± 0.23	0.48 ± 0.29	0.50 ± 0.32
Cu (μg/L)	921.1 ± 170.9	916.0 ± 147.5	850.7 ± 126.4	964.0 ± 209.7	960.2 ± 167.3
Zn (μg/L)	7619.9 ± 1991.1	7194.3 ± 1383.4	6743.7 ± 1394.2	8271.3 ± 2473.8	8414.4 ± 2047.9

Data are presented as *n*(%) or mean ± standard deviation. BMI—body mass index; SBP—systolic blood pressure; DBP—diastolic blood pressure; WBC—white blood cell; RBC—red blood cell; AST—aspartate aminotransferase; ALT—alanine aminotransferase; TC—total cholesterol; TG—triglyceride.

**Table 2:** TNF-α single nucleotide polymorphisms (SNPs) of all participants divided into quartiles according to serum TNF-α levels.

Variable	Total	TNF-α (pg/mL)			
	*n* = 462	<12.84	12.84 ≦ TNF-α <18.99	18.99 ≦ TNF-α <24.34	≧24.34
−238G > A (rs361525)					
GG	424 (91.8)	96 (82.8)	109 (94.8)	109 (94.0)	110 (95.7)
AG	34 (7.3)	16 (13.8)	6 (5.2)	7 (6.0)	5 (4.3)
AA	4 (0.9)	4 (3.4)	0 (0)	0 (0)	0 (0)
−308G > A (rs1800629)					
GG	365 (79.0)	98 (84.5)	95 (82.6)	92 (79.3)	80 (69.5)
AG	89 (19.3)	18 (15.5)	20 (17.4)	24 (20.7)	27 (23.5)
AA	8 (1.7)	0 (0)	0 (0)	0 (0)	8 (7.0)
−857C > T (rs1799724)					
CC	348 (75.3)	94 (81.0)	88 (76.5)	88 (75.9)	78 (67.8)
CT	103 (22.3)	18 (15.5)	27 (23.5)	27 (23.3)	31 (27.0)
TT	11 (2.4)	4 (3.5)	0 (0)	1 (0.8)	6 (5.2)
−863C > A (rs1800630)					
CC	415 (89.8)	92 (79.3)	106 (92.2)	105 (90.5)	112 (97.4)
CA	45 (9.8)	22 (19.0)	9 (7.8)	11 (9.5)	3 (2.6)
AA	2 (0.4)	2 (1.7)	0 (0)	0 (0)	0 (0)
−1031T > C (rs1799964)					
TT	273 (59.1)	72 (62.1)	70 (60.9)	67 (57.8)	64 (55.7)
CT	162 (35.1)	38 (32.7)	41 (35.6)	42 (36.2)	41 (35.6)
CC	27 (5.8)	6 (5.2)	4 (3.5)	7 (6.0)	10 (8.7)

Data are presented as *n* (%).

**Table 3:** Mixed-effect model of the association of covariates with serum TNF-α level.

Parameter	Estimate	SE	*p*
Age (year)	0.24	0.05	<0.0001 *
Gender (male vs. female)	3.76	1.11	0.0008 *
BMI (kg/m^2^)	−0.51	0.13	<0.0001 *
Smoking (yes vs. no)	−0.99	0.96	0.3061
Drinking alcohol (yes vs. no)	3.23	3.17	0.3096
WBC (10^3^/µL)	1.25	0.26	<0.0001 *
Pb (μg/L)	0.23	0.0093	<0.0001 *
Cd (μg/L)	7.65	1.01	<0.0001 *
As (μg/L)	1.23	0.15	<0.0001 *
Se (μg/L)	0.03	0.0051	<0.0001 *
Co (μg/L)	17.22	2.43	<0.0001 *
Cu (μg/L)	0.01	0.0014	<0.0001 *
Zn (μg/L)	0.001	0.0002	<0.0001 *

SE—Standard error; WBC—white blood cell; * Bonferroni’s correction *p* < 0.01.

**Table 4:** Mixed-effect model of the association of covariates and TNF-α SNPs with serum TNF-α level.

Parameter	*n*	Estimate	SE	*p*
Age (year)		0.20	0.05	<0.0001 *
Gender (male vs. female)		1.84	1.13	0.104
BMI (kg/m^2^)		−0.39	0.13	0.002 *
Smoking (yes vs. no)		1.32	0.98	0.180
Drinking alcohol (yes vs. no)		-0.77	3.19	0.808
WBC (10^3^/µL)		0.75	0.27	0.006 *
Pb (μg/L)		0.22	0.01	<0.0001 *
Cd (μg/L)		7.06	0.98	<0.0001 *
As (μg/L)		1.13	0.14	<0.0001 *
Se (μg/L)		0.03	0.01	<0.0001 *
Co (μg/L)		15.56	2.35	<0.0001 *
Cu (μg/L)		0.01	0.001	<0.0001 *
Zn (μg/L)		0.001	0.0002	<0.0001 *
−238G > A(rs361525) *; −308G > A(rs1800629) * −857C > T(rs1799724)*; −863C > A(rs1800630) * −1031T > C(rs1799964)				
G-G/A-A/T-T/C-C/C-C	3	44.98	4.79	<0.0001 *
G-G/A-A/C-T/C-C/C-C	1	28.78	8.07	0.0004 *
G-G/G-A/C-T/C-C/C-C	2	17.87	5.81	0.002 *
G-A/G-G/C-C/C-A/C-C	1	−11.83	8.03	0.141
G-G/G-G/C-C/C-A/C-C	2	−4.82	5.70	0.397
G-A/G-G/C-C/C-C/C-C	5	−6.36	3.66	0.082
G-G/G-G/C-T/C-C/C-C	1	1.99	8.06	0.805
G-G/G-G/C-C/C-C/C-C	12	6.57	2.52	0.009 *
G-G/G-A/C-C/C-A/T-C	1	−4.32	8.02	0.590
G-A/G-A/C-C/C-C/T-C	1	−7.81	8.04	0.332
G-G/G-A/C-T/C-C/T-C	7	6.71	3.15	0.033
G-G/G-A/C-C/C-C/T-C	21	−5.53	1.97	0.005 *
G-A/G-G/C-T/C-A/T-C	1	−12.13	8.04	0.132
G-A/G-G/C-C/C-A/T-C	1	22.23	8.04	0.006 *
G-G/G-G/C-T/C-A/T-C	1	1.05	8.03	0.896
G-G/G-G/C-C/C-A/T-C	4	−6.22	4.06	0.126
G-A/G-G/C-T/C-C/T-C	4	−4.29	4.60	0.350
G-A/G-G/C-C/C-C/T-C	10	9.90	2.75	0.0003 *
G-G/G-G/C-T/C-C/T-C	19	−1.82	2.08	0.380
G-G/G-G/C-C/C-C/T-C	92	−1.57	1.14	0.167
G-G/A-A/C-C/C-C/T-T	4	14.90	4.58	0.001 *
G-G/G-A/C-T/C-A/T-T	1	−10.36	8.05	0.199
G-G/G-A/C-C/C-A/T-T	6	−8.11	3.36	0.016
G-G/G-A/C-T/C-C/T-T	11	2.41	2.62	0.357
G-G/G-A/C-C/C-C/T-T	39	1.61	1.55	0.298
A-A/G-G/C-C/A-A/T-T	1	−11.80	8.04	0.143
G-A/G-G/C-C/A-A/T-T	1	−14.02	8.10	0.084
A-A/G-G/C-C/C-A/T-T	2	−11.64	5.70	0.041
G-A/G-G/C-C/C-A/T-T	8	−12.48	2.96	<0.0001 *
G-G/G-G/C-T/C-A/T-T	6	−5.65	3.94	0.152
G-G/G-G/C-C/C-A/T-T	11	−11.55	2.53	<0.0001 *
A-A/G-G/C-C/C-C/T-T	1	−13.08	8.07	0.105
G-A/G-G/C-C/C-C/T-T	2	−13.68	5.70	0.016
G-G/G-G/T-T/C-C/T-T	8	0.06	3.29	0.986
G-G/G-G/C-T/C-C/T-T	49	−1.04	1.42	0.464
G-G/G-G/C-C/C-C/T-T	123	Reference		

SE—Standard error; BMI—Body mass index; WBC—white blood cell; * Bonferroni’s correction *p* < 0.01.
